# Topical Delivery of Flurbiprofen from Pluronic Lecithin Organogel

**DOI:** 10.4103/0250-474X.51955

**Published:** 2009

**Authors:** M. S. Pandey, V. S. Belgamwar, S. J. Surana

**Affiliations:** Department of Pharmaceutics, R. C. Patel Institute of Pharmaceutical Education and Research, Shirpur-425 405, India

**Keywords:** Pluronic lecithin organogel, flurbiprofen, topical delivery

## Abstract

The purpose of this research is to formulate and evaluate the suitability of pluronic lecithin organogels containing flurbiprofen for topical application. Four formulations were developed using flurbiprofen, lecithin, Pluronic F127, isopropyl palmitate, water, sorbic acid and potassium sorbate were coded as FL1, FL2, FL3 and FL4. All the formulations carried 30% w/w of lecithin phase and 70% w/w of Pluronic phase. The formulated organogels were evaluated for appearance and feel psychorheologically, *in vitro* diffusion study, drug content, viscosity and pH. Release of flurbiprofen from all formulations was monitored via dialysis membrane-70 and Wistar rat skin as a semipermeable membrane into phosphate buffer saline (0.2 M, pH 7.4) using Keshary-Chien diffusion cell. The viscosities of different formulations were determined by using Brookfield Viscometer at 25°. An attempt has been made to explore the potential of pluronic lecithin organogels for topical delivery of flurbiprofen.

Topical drug treatment aims at providing high concentration of the drug at the site of application so as to avoid systemic adverse effects associated with oral administration of drug. Organogel is a vehicle base for the delivery of drugs through the dermal and transdermal route. Organogels are formed by specific kind of small organic molecules, which in many solvents very effectively get self assembled into three dimensional networks there by turning a liquid into a gel[1]. Its micellar structure can contain both water and oil soluble ingredients; it shows excellent drug permeability by diffusion through the lipid intracellular matrix and by slight disorganization of skin. Pluronic and lecithin have become very popular in the topical delivery of drugs. A number of studies have shown that pluronic lecithin organogels (PLOs) have the unique capacity to deliver the drugs through the skin[1,2] and particular medications such as NSAIDs, hormones, antiemetics, opoids and local anesthetics[3] to a specific site when other routes of administration are not viable. Flurbiprofen, a propionic acid derivative is effective antiinflammatory and analgesic recommended in the management of patients with osteoarthritis, rheumatoid arthritis and ankylosing spondylitis. It has a logP/hydrophobicity 4.078, having half-life of 4.7-5.7 h and molecular weight of 244.261 g/mol. These properties make it a potential candidate for topical delivery.

Flurbiprofen and Soya Lecithin were received as gratis samples from FDC Ltd, Mumbai and Phospoholipid GmbH, Nattermannallee, Germany, respectively. Pluronic F-127 was procured from Sigma Aldrich Chemie GmbH, Steinheim, Germany. Isopropyl palmitate, polyethylene glycol-600, sorbic acid and potassium sorbate were supplied by Loba Chemie, Mumbai, India. All other chemicals were of analytical grade and used as received.

The various formulations of PLO[4,5] (Table 1) were developed with different compositions. Oil phase was prepared by mixing soya lecithin and sorbic acid in appropriate quantity of isopropyl palmitate. The mixture was kept overnight at room temperature in order to dissolve its constituents. Aqueous phase was prepared by dispersing weighed amount of Pluronic F-127 and potassium sorbate in cold water. The dispersion was stored in refrigerator overnight for effective dissolution of Pluronic F-127. The next day, active ingredient flurbiprofen was dissolved in polyethylene glycol-600 and mixed with the lecithin-isopropyl palmitate solution; polyethylene glycol-600 was used for solubilization of flurbiprofen. Finally, aqueous phase (70%) was slowly added in oil phase (30%) with stirring at 400 rpm using mechanical stirrer.

**TABLE 1:** FORMULATION COMPOSITION OF PLO

Components	Content (%)	Formulations			
		
		FL_1_	FL_2_	FL_3_	FL_4_
Drug	Flurbiprofen	1.4	3	3	3
	Polyethylene glycol-600	-	10	10	10
Oil phase	Soya lecithin	2	3	5	7
	Sorbic acid	0.2	0.2	0.2	0.2
	Isopropyl palmitate up to	100	100	100	100
Aqueous phase	Pluronic F-127	20	20	20	20
	Potassium sorbate	0.2	0.2	0.2	0.2
	Purified water up to	100	100	100	100

The organogels prepared were evaluated for appearance and feel psychorheologically, drug content and content uniformity at 247 nm in ethanol, pH, viscosity using Brookfield Viscometer and *in vitro* diffusion/permeation using Keshary-Chien diffusion cell. The drug content of different formulations of organogel was determined by taking a standard curve of flurbiprofen in ethanol. For this, accurately weighed 50.0 mg of drug was transferred in a 50 ml volumetric flask, dissolved in ethanol and volume was made up with ethanol. Two millilitres of the solution was pipette out and diluted to 100 ml with ethanol. Then aliquots were further diluted with ethanol to get concentration of 2, 4, 6, 8, 10, 12, 14, 16, 18 and 20 μg/ml. Absorbance were recorded spectrophotometrically and standard curve of flurbiprofen in ethanol was plotted at λ_max_ 247 nm. Further for determining drug content, each formulation (0.5 g) was taken in a 50 ml volumetric flask, diluted with ethanol and shaken to dissolve the drug in ethanol. The solution was filtered through Whatman filter paper No. 42, one ml of the above filtrate was pipette out and diluted to 10 ml with ethanol. The content of the drug was estimated spectrophotometrically by using standard curve plotted at λ_max_ 247 nm.

To test the pattern of release of drug from formulations, *in vitro* diffusion studies[4,6,7] were carried out. The developed formulations were subjected to *in vitro* diffusion through dialysis membrane-70, with molecular weight cut off 12000-14000 D and dehaired abdominal skin of Wistar albino rats was used as a semi permeable membrane using modified Keshary-Chien diffusion cell. The receptor compartment was filled with saline phosphate buffer (0.2 M, pH 7.4) and methanol (90:10). Methanol was added in medium to maintained sink condition. The whole assembly was maintained at 37±1° and receptor solution was stirred with a magnetic stirrer at 100 rpm throughout the experiment. Aliquots (1 ml) were withdrawn at regular interval of 1 h for a period of 8 h and replaced with equal volume of fresh medium equilibrated at 37±1°. All the samples were suitably diluted with medium and analyzed spectrophotometrically at 247 nm for flurbiprofen content.

Viscosities[4,6] of the formulated organogels were determined using Brookfield Viscometer with Spindle no.7 (Model: RV DV-E 230) at 25° with the spindle speed of 10 rpm. The pH of formulated organogels was determined using pH meter. The electrode was immersed in organogels and readings were recorded on pH meter. All the formulations showed drug content in the range of 96-99% indicating uniform distribution of drug throughout the base. The viscosity of all the formulations was found to be in the range 2910-3455 poise. The increase in viscosity with increase in lecithin concentration is might be due to formation of complex network. The results revealed that maximum *in vitro* cumulative percent drug release of flurbiprofen in 8 h was observed from FL2 formulation. Further increase in concentration of lecithin decreased cumulative percent drug release which might be due to extensive formation of network like structure with very high viscosity. Also from the *in vitro* diffusion studies it was found that the permeation of flurbiprofen through dialysis membrane-70 (fig. 1) was more as compared to rat skin (fig. 2). The pH of all the formulations was around the skin pH and found to be in the range of 5.9 to 6.5. All the formulations were smooth in feel and free from grittiness which increases the patient compliance. The data obtained is shown in Table 2. From above studies it may be concluded that formulation FL2, containing 3% lecithin is an effective formulation for topical delivery of flurbiprofen as it showed higher cumulative percent drug release and drug content.

**Fig. 1:**
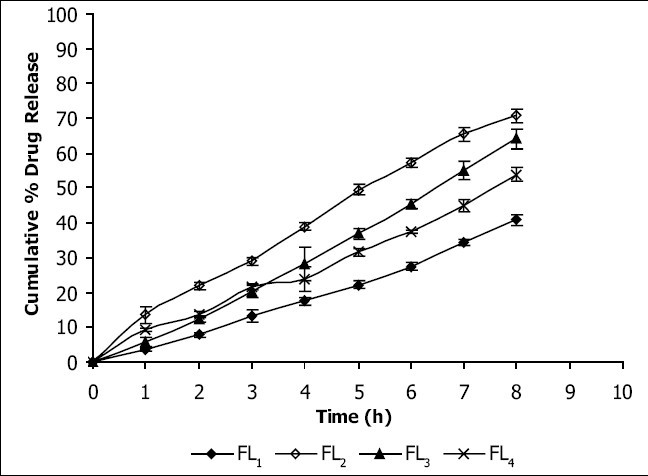
*In vitro* release profile of flurbiprofen through dialysis membrane-70 *In vitro* release rates through dialysis membrane of the four flurbiprofen formulations developed, FL_1_ (-♦-), FL_2_ (-◊-), FL_3_ (-▲-) and FL_4_ (-×-)

**Fig. 2:**
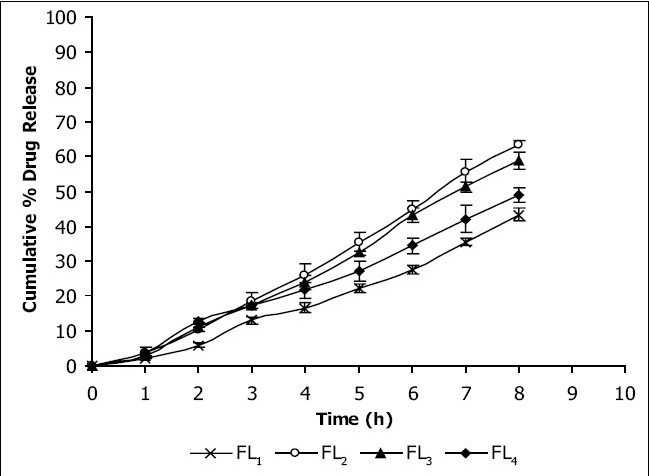
*In vitro* release profile of flurbiprofen through rat skin *In vitro* release rates through rat skin of the four flurbiprofen formulations developed, FL_1_ (-×-), FL_2_ (-◊-), FL_3_ (-▲-) and FL_4_ (-♦-)

**TABLE 2:** DRUG CONTENT, VISCOSITY, pH AND DIFFUSION STUDY OF DIFFERENT FORMULATIONS

Parameters		Formulations			
		
		FL_1_	FL_2_	FL_3_	FL_4_
Drug content (%)		96.58±1.162	98.82±0.594	98.10±0.934	97.48±1.156
Viscosity (poise)		2910±40.63	3167±37.47	3285 ± 45.83	3455±51.64
pH		5.9±0.264	6.33±0.208	6.23 ± 0.351	6.467±0.252
Diffusion	Dialysis	40.82±1.676	70.83±1.897	64.12 ± 2.72	53.78±2.016
study (%)	membrane-70 Rat skin	43.26±1.847	63.47±0.972	58.92 ± 2.395	49.03±2.18

Each value is mean±SD (n=3)
